# Constraints faced by urban poor in managing diabetes care: patients’ perspectives from South India

**DOI:** 10.3402/gha.v6i0.22258

**Published:** 2013-10-03

**Authors:** Upendra Bhojani, Arima Mishra, Subramani Amruthavalli, Narayanan Devadasan, Patrick Kolsteren, Stefaan De Henauw, Bart Criel

**Affiliations:** 1 Institute of Public Health, Bengaluru, India; 2 Department of Public Health, Institute of Tropical Medicine, Antwerp, Belgium; 3 Department of Public Health, Ghent University, Ghent, Belgium; 4 Health, Nutrition and Development Initiative, Azim Premji University, Bengaluru, India

**Keywords:** access to care, diabetes, chronic illness, slum, healthcare service, patients’ perspective

## Abstract

**Background:**

Four out of five adults with diabetes live in low- and middle-income countries (LMIC). India has the second highest number of diabetes patients in the world. Despite a huge burden, diabetes care remains suboptimal. While patients (and families) play an important role in managing chronic conditions, there is a dearth of studies in LMIC and virtually none in India capturing perspectives and experiences of patients in regard to diabetes care.

**Objective:**

The objective of this study was to better understand constraints faced by patients from urban slums in managing care for type 2 diabetes in India.

**Design:**

We conducted in-depth interviews, using a phenomenological approach, with 16 type 2- diabetes patients from a poor urban neighbourhood in South India. These patients were selected with the help of four community health workers (CHWs) and were interviewed by two trained researchers exploring patients’ experiences of living with and seeking care for diabetes. The sampling followed the principle of saturation. Data were initially coded using the NVivo software. Emerging themes were periodically discussed among the researchers and were refined over time through an iterative process using a mind-mapping tool.

**Results:**

Despite an abundance of healthcare facilities in the vicinity, diabetes patients faced several constraints in accessing healthcare such as financial hardship, negative attitudes and inadequate communication by healthcare providers and a fragmented healthcare service system offering inadequate care. Strongly defined gender-based family roles disadvantaged women by restricting their mobility and autonomy to access healthcare. The prevailing nuclear family structure and inter-generational conflicts limited support and care for elderly adults.

**Conclusions:**

There is a need to strengthen primary care services with a special focus on improving the availability and integration of health services for diabetes at the community level, enhancing patient centredness and continuity in delivery of care. Our findings also point to the need to provide social services in conjunction with health services aiming at improving status of women and elderly in families and society.

Globally, over 371 million adults had diabetes in the year 2012, and four out of five adults with diabetes lived in low- and middle-income countries (LMIC) (1). Southeast Asia accounts for nearly one-fifth of diabetes patients in the world. India alone has 63 million diabetes patients (1). In most countries, the prevalence of diabetes, its risk factors, and adverse outcomes is higher among poor adults, with recent evidence suggesting a similar trend in India (1–3, unpublished data).

Despite the huge burden, diabetes care remains suboptimal in LMIC. Studies from India reveal a huge gap between the recommended and actual diabetes care, resulting in poor health outcomes (4–7). A study from South India revealed that 20.1% of type 2 diabetes patients did not receive any treatment while 71.2% had poor glycaemic control (5). An earlier study from the region showed that only 25% of type 2 diabetes patients adhered to prescribed medication (6). Another study from Delhi revealed poor glycaemic control; 79.4% of patients did not adhere to the prescribed medication and 41.4% did not visit a primary care provider in the previous year (4). One multicentre study in India revealed similar results (7).

Effective management of diabetes, or of any chronic condition, requires coordinated efforts from the healthcare team, patients, families, and other partners in the local community, including a favourable environment that allows for and promotes a coordinated response (8). While there are some anthropological and sociological studies capturing patients’ perspectives, such studies from LMIC, including India, are conspicuously absent. In fact, poor treatment outcomes are commonly attributed to a lack of ‘knowledge’ and poor treatment ‘compliance’ by patients. Furthermore, the specificity of context that plays a major role in shaping social interpretation and response to an illness does not allow for a ready transfer of available knowledge in high-income countries to other contexts. A few researchers have studied socio-cultural issues in LMIC while exploring associations between stress, depression and diabetes, suggesting the need to complement the biomedical approach for diabetes management with a psychosocial approach (9–12). However, there is a lack of in-depth studies exploring patient experiences in managing diabetes care from a health service perspective.

Patients’ experiences could highlight how specific constraints or facilitators to access healthcare are constructed within the lived realities of the patients. Such insights are crucial in reforming health services so that care systems would become more patient centred by enhancing patients’ satisfaction and participation in decision making, treatment compliance, and other parameters of quality of care (13). These insights may also help to decipher seemingly contrasting observations made by some surveys in India. For example, glycaemic control and utilisation of preventive services are poor among relatively wealthy patient groups in Indian metropolitan cities, despite greater awareness about diabetes and the availability of health facilities in these cities (4, 5).

In this article, we aim to better understand constraints faced by patients from urban slums in South India in managing care for type 2 diabetes. We also report the suggestions of these patients to improve diabetes care.

## Methods

We conducted a cross-sectional study using in-depth interviews with self-reported type 2 diabetes patients. We used a phenomenological approach to explore and understand patients’ detailed accounts of living with disease, healthcare-seeking behaviour, and expectations from health services. A phenomenological approach, widely used in medical sociology and anthropology, enables a researcher to understand the embodied and lived experiences of patients (14). These accounts also offer a window into the local community's world of shared meanings around health and illness in general and diabetes in particular. This approach has been used in this study to understand patients’ lived experiences of chronic illness conditions focusing on diabetes.

We conducted this study in Kadugondanahalli (KG Halli), a poor urban neighbourhood with a designated slum area in a metropolitan capital (Bengaluru) of Indian state of Karnataka. KG Halli has a population of over 44,500 people. Over 75% of population lives below two USD (INR 110) a day. Most people earn their living through manual labour, small businesses, and factory jobs. Majority (68.7%) of the population is Muslim. Hindus (21%) and a small minority of Christians (10.3%) are the next most common religious groups. KG Halli is also a home to migrants from the neighbouring states and at least three languages are commonly spoken in the area. KG Halli has two government health centres that provide free care and at least 32 private facilities providing care on a fee-for-service basis. The private healthcare delivery sector is highly utilised and unregulated. Most private facilities are single-doctor clinics with trained or untrained healthcare providers who offer care in different systems of medicine (mainly modern medicine, Ayurveda and Unani). This pluralistic nature of the healthcare delivery system, with several healing traditions (especially Ayurveda, Yoga, Naturopathy, Unani, Siddha, Homeopathy) operating next to modern medicine, is a major feature of the Indian health system (15). There are four private hospitals in the area with varying capacities and many private pharmacies and laboratories.

This study followed an earlier survey delineating a high burden of diabetes among KG Halli residents (16, 17). We selected type 2 diabetes patients with the help of the four community health workers (CHWs) who have been working in KG Halli for the past 3 years as part of the Urban Health Action Research Project. The aim of this project is to improve quality of healthcare for KG Halli residents by working with healthcare providers, health authorities, and residents of KG Halli. The CHWs are women from KG Halli and adjoining neighbourhoods. They received training on basic health issues and they make periodic visits to households in KG Halli creating awareness on health issues and linking patients with appropriate healthcare resources in the area under supervision of the project staff. As a result, CHWs have build rapport with patients and residents in KG Halli and they maintain a diary about their encounters with patients. After explaining the purpose of the study, CHWs were asked to suggest potential respondents residing in their respective sub-areas. Use of CHWs as informants enabled the identification of patients willing to share their personal experience and allowed to respect minimal sampling criteria (i.e. patients from both sexes presenting different stages of disease and treatment). The purposive sampling of respondents continued until gross data saturation was achieved and qualitative themes became repetitive.

We opted for informed oral consent due to the low literacy level and apprehensions about signing documents among KG Halli residents. The consent process and outcome was audio-recorded. There were no refusals. Based on respondents’ language preferences, the first author conducted interviews in *Hindi* and *Urdu*, and the third author (accompanied by the first author) conducted interviews in *Kannada* and *Tamil*. Both interviewers had formal training in qualitative research. The first author developed an initial interview guide providing general guidance on approaching diabetes patients and exploring broad enquiry domains (Table 1), including specific probes based on previous knowledge. The other authors reviewed this guide. Subsequent to the initial interviews, the interview guide was refined, mainly in terms of adding further probes. The interviewers explored the patients’ experiences of living with and seeking care for diabetes in a contextually sensitive and non-judgemental manner allowing patients to narrate on their own terms and move beyond the broad enquiry topics. All interviews were tape-recorded and took place in the patients’ residence. Interview duration ranged from 50 to 90 min. A professional transcriptionist translated and transcribed interviews into English. These transcripts were then verified and edited by the first and the third authors.

**Table 1:** Broad enquiry domains explored in the interviews

Onset and interpretation of illness
Experiences of seeking healthcare
Perceptions about risks, precautions, illness management, and complications
Suggestions for/expectations from healthcare services

The first author started analysing data concurrently with data collection without using a particular model but considering a broad enquiry of understanding how patients perceive, make sense of and manage their illness. Data were organised and initially coded using the NVivo software. These initial codes with emerging themes were periodically discussed among the research team at the KG Halli Urban Health Action Research Project, including the second author (a senior medical anthropologist) to understand the patients’ reasoning. Over time, through iterative processes, these themes were refined. Relationships between and across themes were explored using the mind-mapping tool, MindNodeLite to uncover the larger story behind patients’ narration of their everyday experiences. This story indicated several constraints that patients face at different yet interrelated sites relating to gender, financial hardship, generational conflict and attitudes of health providers. The results section follows these categories in describing and interpreting the patients’ experiences. The potential associations between respondents’ demographics and the themes were also explored.

This study received approval from the Institutional Review Board at the Institute of Tropical Medicine, Antwerp, Belgium, and the Institutional Ethics Committee at the Institute of Public Health, Bengaluru, India.

## Results

We conducted 16 in-depth interviews with patients. Table 2 provides demographic characteristics of respondents. We now describe the results using the major themes that defined constraints in accessing healthcare. Table 3 provides a summary of these themes.

**Table 2:** Profile of the respondents

Respondent number	Sex	Age (years)	Approximate duration (years) since diagnosis of type 2 diabetes	Comorbidity	Occupation
R1	Woman	21	2.5	–	Homemaker
R2	Woman	35	7	Hypertension	Homemaker
R3	Woman	45	3	Hypertension	Homemaker
R4	Woman	52	8–9 months	Hypertension	Homemaker
R5	Woman	60	9	Hypertension	Homemaker
R6	Woman	65+	3–4	Hypertension	Homemaker
R7	Woman	55	8	Hypertension	Homemaker
R8	Man	48	15	Heart problem	Self-employed (small business)
R9	Man	54	15	Heart problem	Not working
R10	Man	46	8	–	Semi-skilled labour (tile cleaning)
R11	Man	38	10	Kidney problem	Skilled labour (plumbing)
R12	Man	43	8	Hypertension	Skilled labour (tailoring)
R13	Man	55	11	–	Skilled labour (driver)
R14	Man	65	10	Hypertension	Not working
R15	Man	48	2	–	Not working
R16	Man	44	6	–	Skilled labour (driver)

**Table 3:** Dominant themes defining constraints to diabetes care

**Struggling to make both ends meet**
***Diabetes taking a lesser priority***
*I think twice before buying tablets worth ten rupees [approximately 0.2 USD]. I can use those ten rupees to buy a coconut and prepare chutney* [paste] *and serve it with food to them* [family members]. (R3, woman 45 years)
***Compromised care***
*He* [doctor] *told me to take one tablet per day but it is costly and so I take half a tablet per day*. (R4, woman 52 years)
***Self-adjustments of medication***
*I take it* [medication] *only when I feel I have a problem* [frequent urination]. (R13, man 55 years)
**On being ‘woman’ and ‘elderly’**
***Gender-based family roles***
*They* [staff at a government secondary hospital] *asked me to get admitted. I told them that I have a very young child to take care of and came back home. … I could not go again. It will be a problem if I get admitted in the hospital, leaving the baby at home*. (R1, woman 25 years)
***Nuclear family structure***
*If someone takes* [me to health facility], *I go. I can't see properly so it is difficult to cross that road* [to reach the government health centre]. *Yesterday, I was out and not able to see anything and bumped into someone*. (R6, woman 65+ years)
***Inter-generational conflicts***
*My son is able to buy medicines for me, but he probably feels that it is a waste of money as I am old*. (R14, man 65 years)
**Providers’ attitudes and communication**
***Inadequate communication***
*He* [doctor] *does not explain anything. As soon as I go there, he will write a prescription, take his fees and send us away …*. *Only if he tells us not to eat this, or to eat only that, we will know about it. But if he himself doesn't tell us, what will we know? We are uneducated, so we will simply sit quietly*. (R2, woman 35 years)
***Negative attitudes***
*We would have left the children at home, and at the hospital we will keep thinking about home, family etc. that we would have left behind and come to the hospital …. When we go to the hospital in this state of mind, they should talk properly to us and tell us that this is the problem* …. (R8, man 48 years)
***About ‘good’ doctors***
*By the grace of Allah, after we started going to this doctor, the sugar level is under control and the doctor also explains everything properly. Even if we have to spend a little more money, it gives me peace of mind*. (R5, woman 60 years)
**Approaching the health system**
***Fragmented primary care***
*At XXX* [a government centre], *they say that I have to get* [my] *blood checked at some other place* [private laboratory], *and take the report to them to get the medication. I do not want it in that manner …. If you want to help poor people, all the facilities should be there at one place*. (R7, woman 55 years)
***‘Free’ health camps***
*One more thing is, to become popular, the politicians put up boards and organise camps for eye testing and diabetes, once in a year* …. *There, they* [doctors] *check, and tell us to come to their hospitals for medication* …. *If we go there* [hospitals], *they will charge a minimum of one thousand rupees* [approximately 18.2 USD] *for the treatment*. (R3, woman 45 years)
***Tertiary care***
*The treatment there* [a tertiary government hospital] *is free of cost, but we will be made to do a lot of running around. For this one problem* [kidney problem]*, I was running around for eight days…. If you go to a doctor once, he will not be there when you go there for the second time. There will be some other doctor. This doctor will ask us to get some other test done. If each doctor says something different, what shall I do?* (R9, man 54 years)

### Struggling to make both ends meet

Financial hardship affected the respondent's daily living, including how they managed diabetes.

#### Diabetes taking a lesser priority

Earning enough money to sustain daily living was a major concern among the families of respondents. The families of eight of the respondents were in debt at the time of the study. Therefore, feeding the family and repaying loans was a higher priority than the diabetes management.

I think twice before buying tablets worth ten rupees [approximately 0.2 USD]. I can use those ten rupees to buy a coconut and prepare chutney [paste] and serve it with food to them [family members]. (R3, woman 45 years)

People like us are fed up repaying interest on the loan taken. We do not know when we will be able to come out of this, and peacefully and happily drink ganji [rice porridge]. (R10, man 46 years)

Patients often drew upon experiences of other members of their community, highlighting the collective nature of the experience.

Not just me, everybody here has problems …. (R3, woman 45 years)

#### Compromised care

The financial constraints appeared to be a major barrier in accessing chronic illness medication that should be taken for years or a lifetime. Three respondents were not on medication while six respondents were not taking medication on a regular basis.

If I have money, I will buy medicines. If I do not have money, I will just keep silent. (R9, man 54 years)

Some patients reduced their medication dosage so that medication would last longer while one patient reported mixing modern medication with Ayurveda remedies to reduce the overall cost.

He [doctor] told me to take one tablet per day but it is costly and so I take half a tablet per day. (R4, woman 52 years)

I take these [allopathic] medication and Ayurveda medication for two weeks alternatively to reduce spending [on medication]. (R11, man 38 years)

Some patients, who had diabetes for three or more years, explained how, based on their bodily experience, they would come to know when their blood sugar and blood pressure was high or low. These patients would accordingly decide when to take medication or alter the prescribed dosage of their medication without seeking professional advice. Often the development of co-morbid illness or diabetes complications made patients to start medication.

I take it [medication] only when I feel I have a problem [frequent urination]. (R13, man 55 years)

After suffering the heart attack, I started taking medication regularly. (R8, man 48 years)

### On being ‘woman’ and ‘elderly’

#### Gender roles

Women were primarily seen as homemakers who cook, perform household chores, host visitors, and care for the children. None of the woman respondents were engaged in paid employment. These roles restricted these women from going out for a walk or seeking care from a preferred health facility, especially distant facilities.

They [staff at a government secondary hospital] asked me to get admitted. I told them that I have a very young child to take care of and came back home. … I could not go again. It will be a problem if I get admitted in the hospital, leaving the baby at home. (R1, woman 25 years)

Household power dynamics implied that women were subservient to the rest of the family. The management of diabetes, especially non-medical treatment, including dietary modification and exercise, were prioritised for men.

In this age of rising costs, I will have to eat whatever food is left over …. I prepare rice for children but if it is left over, I will have to eat it. My husband will not eat it. (R4, woman 52 years)

#### Family structure and inter-generational conflicts

In KG Halli and in urban India, in general, smaller nuclear families are becoming common. In these families, the elderly live alone or with unmarried children and are often financially dependent on their married sons. This structure limits family support that is generally available in traditional extended families for elderly adults.

If someone takes [me to health facility], I go. I can't see properly so it is difficult to cross that road [to reach the government health centre]. Yesterday, I was out and not able to see anything and bumped into someone. (R6, woman 65+ years)

Inter-generational conflicts were common and resulted in strained relationships between daughters-in-laws and mothers-in-law and between elderly parents who are financially dependent on their sons.

My son is able to buy medicines for me, but he probably feels that it is a waste of money as I am old. (R14, man 65 years)

What happens, she [daughter-in-law] cooks and sends food to me. Now what to say to her? [Pause and sigh]. If my daughter would be there, I would not have to even tell her. She would have cooked accordingly [without adding sugar and salt]. My daughter-in-law stays nearby but does not take care [of me]. At my house, a neighbour comes to wash my clothes and will make me a bath. (R6, woman 65+ years)

### Providers’ attitudes and communication

#### Inadequate communication

Patients expressed major concerns about the attitude of healthcare providers and inadequate communication during a consultation. Poor patients hesitated to ask lingering questions about their health conditions and management.

He [doctor] does not explain anything. As soon as I go there, he will write a prescription, take his fees and send us away …. Only if he tells us not to eat this, or to eat only that, we will know about it. But if he himself doesn't tell us, what will we know? We are uneducated, so we will simply sit quietly. (R2, woman 35 years)

Some patients found that doctors were rude and lacked consideration for the patient's social and emotional circumstances. Patients expected the doctors to provide reassurance to patients and families rather than creating fear or blame.

When we went to XXX [a government tertiary hospital], the doctor said there is a problem and nothing can be done. An operation is not required as the patient is very weak. They also created fear in our mind by saying that all human beings have to die one day …. We would have left the children at home, and at the hospital we will keep thinking about home, family etc. that we would have left behind and come to the hospital …. When we go to the hospital in this state of mind, they should talk properly to us and tell us that this is the problem … (R8, man 48 years)

Patients expressed these concerns about doctors in both the public and private sectors and across the different levels of the healthcare system.

#### On ‘good’ doctors

Some patients immediately recounted their interactions with ‘rare’ doctors in the private sector who exhibited empathy and sympathetic listening. These characteristics of the doctors were highly valued by patients who preferred to seek care from these doctors, even at the cost of longer waiting times or relatively higher fees.

By the grace of Allah, after we started going to this doctor, the sugar level is under control and the doctor also explains everything properly. Even if we have to spend a little more money, it gives me peace of mind. (R5, woman 60 years)

### Approaching the health system

The patients described difficulties accessing care on a long-term basis for diabetes and other chronic conditions at multiple levels of the healthcare system.

#### Fragmented primary care

At the primary care level (government health centres and private clinics), patients had to visit multiple locations to receive care for a single medical encounter. The local government health centre did not offer blood sugar examination, and most private clinics lacked laboratory and pharmacy services within their facilities.

At XXX [a government centre], they say that I have to get [my] blood checked at some other place [private laboratory], and take the report to them to get the medication. I do not want it in that manner …. If you want to help poor people, all the facilities should be there at one place. (R7, woman 55 years)

#### ‘Free’ health camps

Often, patients were diagnosed with diabetes at health camps or community outreach programmes, which were generally free of cost. However, such services were mainly *ad-hoc* diagnostic initiatives that were not linked to long-term, free healthcare services. In fact, some patients noted that private hospitals used these ‘free’ camps to market their services and recruit patients while politicians hosted these camps during election time to appease voters.

One more thing is, to become popular, the politicians put up boards and organise camps for eye testing and diabetes, once in a year …. There, they [doctors] check, and tell us to come to their hospitals for medication …. If we go there [hospitals], they will charge a minimum of one thousand rupees [approximately 18.2 USD] for the treatment. (R3, woman 45 years)

#### Tertiary care

Patients visited these facilities when they experienced complications or when primary care providers referred them there. Common concerns about these large hospitals, which were mainly public sector facilities, included the need for repeat visits, long wait times, poor availability of doctors, frequent rotation of doctors, lack of coordination between doctors resulting in different opinions, and high opportunity cost due to long travel time and forgone wages.

The treatment there [a tertiary government hospital] is free of cost, but we will be made to do a lot of running around. For this one problem [kidney problem], I was running around for eight days …. If you go to a doctor once, he will not be there when you go there for the second time. There will be some other doctor. This doctor will ask us to get some other test done. If each doctor says something different, what shall I do? (R9, man 54 years)

## Discussion

Our study provides the perspectives of patients as they sought diabetes care. Despite an abundance of healthcare facilities in the vicinity, the patients faced several barriers to care. These barriers included financial and familial constraints, the negative attitudes of providers, inadequate communication, and the limited and fragmented nature of the existing healthcare system.

For more than three decades, financial constraints have remained the second most common reason for not seeking healthcare in India (18). A recent study from Delhi revealed that poor diabetes patients avoided or delayed healthcare due to financial constraints (9). An earlier survey in KG Halli revealed that 69.6% of families incurred out-of-pocket expenses for outpatient care for chronic conditions, nearly doubling the poverty rate every month (17). The largest share of out-of-pocket spending on healthcare, in general, and for chronic conditions, in particular, has been on medication (19, 20). Our findings reinforce this fact explaining the reasons: the unavailability of medication and diagnostics within government primary care facilities and the need for patients to visit different facilities for different components of care within the expensive private sector. Hence, integrating these services in one location in the public sector and controlling costs in the private sector could help reduce the cost of diabetes care for patients. In the interim, there is also a need to provide financial protection to patients against a huge impoverishing out-of-pocket healthcare costs.

The ‘free’ diabetes camps that are often organised in poor urban areas are ethically questionable, as these initiatives lead to diagnose people with diabetes without offering them free or subsidised follow-up care. In fact, mainly private healthcare providers use these camps as a marketing strategy where they bank upon turning an undiagnosed burden of diabetes into new patients for their clinics/hospitals. Political leaders, with the support from healthcare providers, consider these camps as a convenient tool to appeal to masses in pre-election times. These so-called charitable or sympathetic activities are bluntly overlooking people's life circumstances and the many constraints diabetes patients face in their search for proper care. We suggest that such camps provide subsidised follow-up care or, at the very least, that they facilitate patients’ access to affordable healthcare in their surroundings.

Considering the overall financial struggle for daily existence that defines the lives of the urban poor, the measures to provide financial protection for health to this group need to be complemented and integrated with broader social protective measures that promote livelihoods and other social services. In this view, the linkages envisaged between the forthcoming National Urban Health Mission, a flagship programme by the Government of India aimed at enhancing health of urban poor, and the already existing schemes addressing issues of urban development, employment, and child development need to be contextualised and strengthened (21).

The self-prescription and adjustment of medication dosages based on bodily experience by patients in our study mainly appears to be a coping response to limited access to affordable medication. This practice also reflects an acquired understanding of how one's body reacts to a change in the status of a disease/medication. Studies exploring the perspectives of patients with diabetes and epilepsy reveal that patients often assert their control over their disease by deviating from the prescribed dosage and self-adjusting their medication (22, 23). Our study reflected this notion but the prominence of financial constraints in shaping medication use was evident in our study. It is important that healthcare providers attempt to explore and understand these varied notions that define medication practices by patients rather than merely labelling patients as ‘non-compliant’.

Doing this requires that providers spend time communicating with patients to understand their perspectives. However, in our study, patients felt that doctors hardly gave time to patients and that the consultation was not comforting to patients. Other studies in India have shown that the average consultation time in primary care settings has remained pitiably short (from 2.5 min to a maximum of 5 min) (24–26). Patients were quite dissatisfied that doctors did not explain or discuss care with them (24–27). Clearly, our study points to the need for providers to understand a patient's context and perspective and add a human dimension to medical care. That is, care should be more patient centred.

In the absence of a functional social welfare system, the family remains a major source of support for the elderly in India and many other LMIC. However, the transition from a traditional extended family structure to a nuclear family structure has isolated the elderly, reducing their access to healthcare (28, 29). In 2004, 20.2% of elderly adults in India had no family support (i.e. living without a son or daughter under the same roof) (28). Sridhar and colleagues highlight the impact of the nuclear family structure showing that the majority of diabetes patients primarily depended on their spouses and, to a much lesser extent, their children, for support to cope with their diabetes (10, 11). The demographic transition, with rapidly increasing numbers of elderly in India – over 100 million in 2012 (30) – will only make matters worse. These observations reiterate the need to enhance outreach and community-based health services in conjunction with provision of social services for the elderly to improve their health.

Another important contribution of our study is that it demonstrates the gendered nature of diabetes care in India. Gender-based family roles negatively affected women's access to healthcare by restricting their mobility and autonomy. The role of gender is widely recognised to affect health and healthcare in general, including treatment of chronic conditions (31). A few studies in India that assessed depression, anxiety, energy levels, and positive wellbeing in patients with diabetes revealed that men fared better than women (12, 32). Our study findings could help health providers to better understand patient practices and accordingly tailor their advice and treatment. These findings also imply a need for non-health interventions that contribute to promote social norms enhancing women's status and autonomy within families and society.

We plan to use our study findings to formulate health service interventions that might improve the quality of diabetes care in KG Halli. A limitation of our study is that while use of CHWs helped to locate respondents with desirable profile, we might have missed patients who were out of reach of CHWs. Furthermore, while the position of interviewers (doctor and staff of the ongoing project in the area) facilitated familiarity and connect with the patients, it would have affected the patients narratives that tend to emphasise ‘medical’ problems and solutions (that a ‘doctor’ or ‘health project’ can understand/address). Our findings could not be readily generalised to all the urban poor areas in India or in the region. However, our study highlights the need to understand the patients’ experiences and perspectives in studying diabetes care and offer analytical guidance while studying such groups in India and in the region.

## Conclusions

Our study shows that, despite the abundance of healthcare facilities in the vicinity, diabetes patients in poorer urban areas face several constraints in accessing formal healthcare services. These constraints included financial barriers, the negative attitudes and inadequate communication of healthcare providers, and the limited and fragmented nature of the existing healthcare services. Our study demonstrated a gender disadvantage for women in diabetes care. The prevailing nuclear family structure and inter-generational conflicts limited support and care for the elderly.

There is need to strengthen primary care services with special focus on improving availability and integration of health services for diabetes at the community level, promoting patient-centred care, and improving continuity in delivery of diabetes care. Our findings also point to the need to provide social services in conjunction with health services aiming at improving status of women and elderly in families and society.
